# Pulpo-Periodontal Regeneration: Management of Partial Failure Revascularization

**DOI:** 10.1155/2017/8302039

**Published:** 2017-09-17

**Authors:** Said Dhaimy, Sara Dhoum, Hind Amarir, Hafsa El Merini, Sellama Nadifi, Amal El Ouazzani

**Affiliations:** ^1^Department of Odontology Restorative Endodontics, Faculty of Dentistry, Hassan II University of Casablanca, BP 5696, Casablanca, Morocco; ^2^Genetics Laboratory, Faculty of Medicine and Pharmacy, Hassan II University of Casablanca, Casablanca, Morocco

## Abstract

The aim of this work is to present a case of management of an open apex on a lower molar by using tissue engineering, with two endodontic procedures in the same tooth. We had to resort to pulp regeneration on the distal root and apexification with MTA on the mesial roots after the failure of regenerative therapy on those ones. The management consisted in scheduling regular follow-ups combined with X-rays. After 24 months, the radiological control has shown pulpo-periodontal regeneration associated with walls thickening and distal root elongation and periapical ad integrum healing.

## 1. Introduction

The treatment of necrotic immature permanent teeth comes up with some difficulties. Not only is the root canal system often hard to clean completely, but also the thin dentin walls increase the risk of a subsequent root fracture as well [1].

Historically, acceptable long-term results are obtained through apexification procedures using calcium hydroxide [1, 2].

However, because of the multiplicity of renewal sessions, the length of the procedure, and the alteration of the mechanical properties of the dentin, other treatment strategies are proposed using the Mineral Trioxide Aggregate (MTA) to generate an artificial apical barrier. In fact, it is an excellent predictable alternative to address these issues by creating a biocompatible apical plug in a single visit [3]. This procedure can manage the biological factor but without solving the problem of the root fragility.

Lately, regenerative endodontic procedures have been used to treat immature permanent teeth with infected or noninfected necrotic pulps, thus becoming an innovative conservative option and an alternative treatment of immature permanent teeth. Its primary goal is to eliminate clinical symptoms and resolution of apical periodontitis, while increasing the thickening of the canal walls and continued root development are secondary goals in those considerations [4].

This procedure uses the full potential of tissue regeneration through stem cells, leading to the completion of root edification, thus decreasing the risk of fracture due to the fragility of the immature root [5–7].

Regenerative endodontics is defined as a biologically based procedure designed to physiologically replace damaged tooth structure, including dentin and root structures, and the pulp-dentin complex [8].

## 2. Clinical Case

An eighteen-year-old patient showed up in consultation with a tumefaction at the lower right cheek.

### 2.1. Clinical Examination

**Figure 1:**
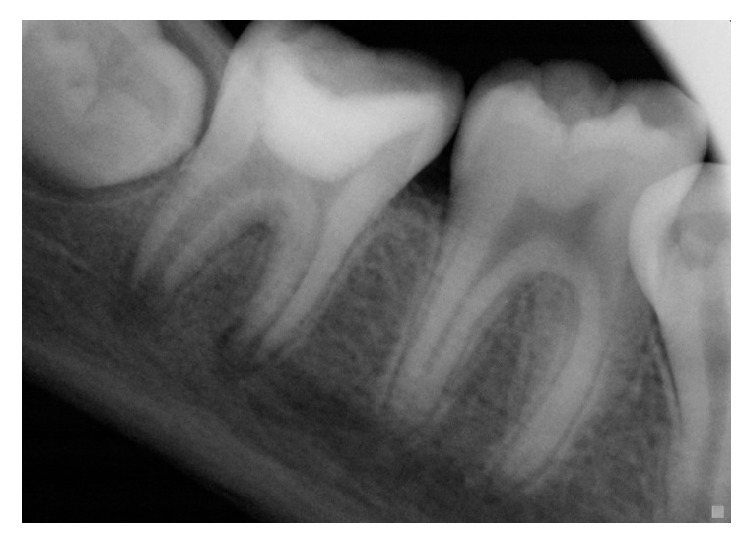
Initial radiography: tooth 47 with radiolucent images at the mesial and distal roots.

Clinical examination showed a buccal filling at the right mandibular molar area and a temporary restoration on #47; vitality tests were all negative (Figure 1).

### 2.2. Diagnosis

Diagnosis of chronic apical periodontitis was made.

It has been decided, with the patient's consent, to use regenerative procedure of the pulp.

The followed protocol was the one established by the American Association of Endodontics [8].

## 3. First Appointment

**Figure 2:**
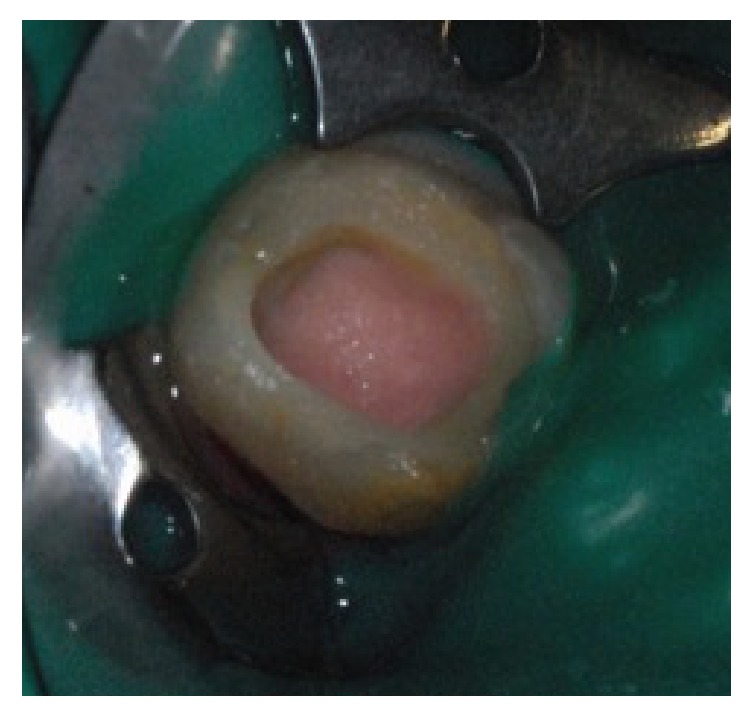
Endodontic filling with the antibacterial medication.

- Local anesthesia without vasoconstrictor (to promote pulp bleeding).
- Dental dam.
- Removal of the temporary filling and correction of the access cavity.
- Determination of the working lengths, using an electronic apex locator Root ZX II (J.Morita Europe, Dietzenbach, Germany) verified with radiography.
- A slight mechanical preparation was performed to clean the root canal using H files assisted by irrigation with 1% sodium hypochlorite, using an endodontic needle introduced at WL-1 mm, followed by ultrasonic activation to improve the debridement of the root canal.
- Preparation of the antibiotic paste: 1.5 MIU Spiramycin pill and 250 mg Metronidazole were ground and mixed with distilled water (Figure 2).
- The antibacterial medication is placed in the canals to the apex using a mouth spatula and lentulo.
- A temporary restoration using Cavit™ has been set up for 2 weeks.

## 4. Second Appointment (After 3 Weeks)

**Figure 3:**
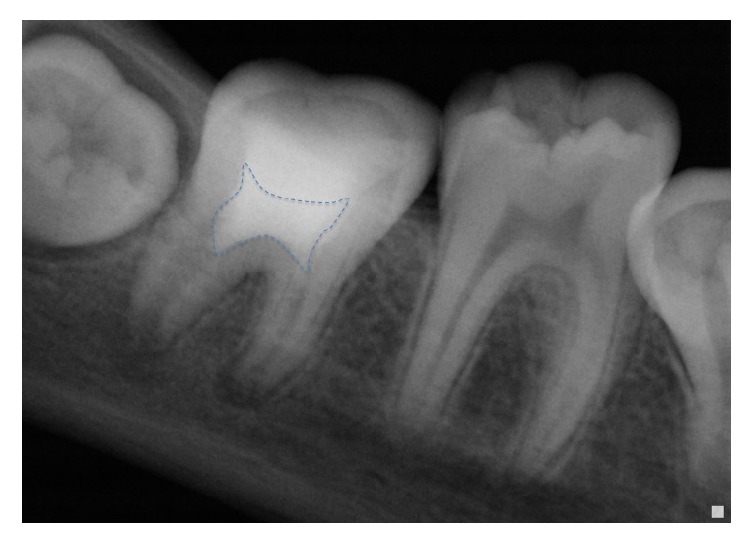
Control radiograph after capping with MTA.

- Removal of the temporary restoration under isolation with dental dam.
- Debridement of the antibiotic paste that was made using endodontic files under copious and gentle irrigation with 1% sodium hypochlorite.
- Coating of the inner walls of the access cavity with adhesive to avoid dyschromia associated with this procedure by preventing blood infiltration in dentinal tubules.
- Initiation of the bleeding by a controlled instrumental overtaking at the apical zone.
- Capping of the blood clot with MTA (Figure 3).
- Protection of the MTA with Glass Ionomer cement as an intermediate base for subsequent final restoration with composite.
- An antibiotic prescription: 3 MIU Spiramycin + 500 mg Metronidazole for 7 days and a paracetamol-based painkiller 1500 MG/Day for 3 days.

## 5. Follow-Up

**Figure 4:**
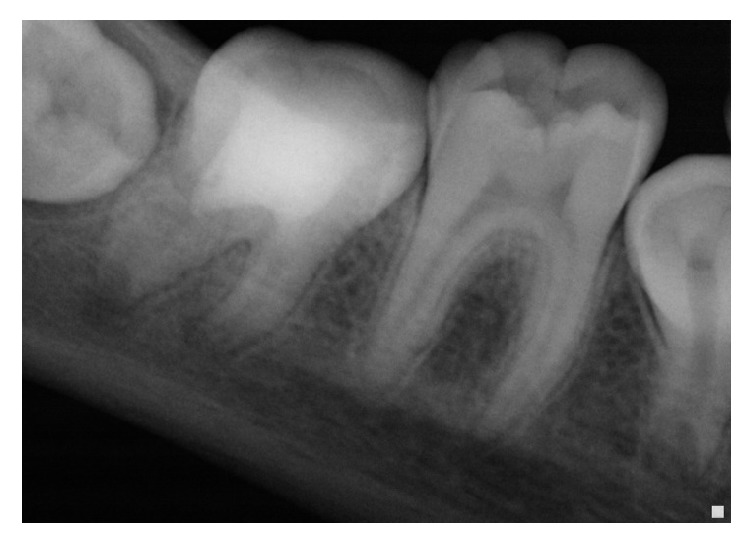
Radiograph after 3 months' recall: reduction of the radiolucent image at the distal root.

**Figure 5:**
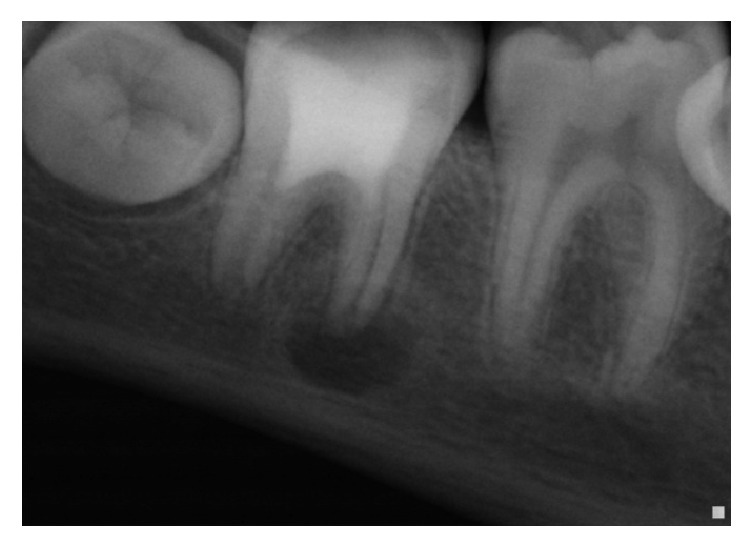
Radiograph after 10 months' recall: enlargement of the radiolucent image at the mesial roots.

- One-week recall: we have noticed a loss of pain on palpation and percussion.
- Control radiographs were performed after 3 months and 10 months (Figures 4 and 5).

The analysis of these radiographs has shown the following.

*At the Distal Root*.

- Progressive reorganization of the periodontal space.
- Reduction of the initial periapical image.

*At the Mesial Roots*. After 10 months' recall (Figure 5), the radiograph has shown a failure of the revascularization therapy, a larger radiolucent image than the initial one, and a sign of recurrent periapical infection.

Believing in the tissue preservation principle and the potential of the periodontal regeneration, we have chosen to retreat the mesial roots with classical apexification therapy using MTA, thus retaining the therapeutic success of the distal root.

**Figure 6:**
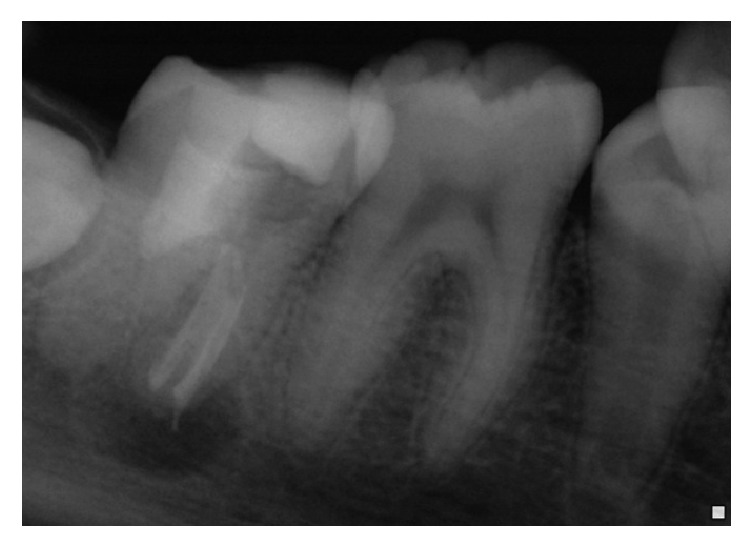
Filling of the mesial roots with MTA.

After conducting a mini access cavity, we have reached the MTA layer; with the assistance of ultrasonic inserts, the MTA in the cervical mesial zone was selectively removed to access the mesial canals. Both of the mesial canals were prepared by the coronoapical technique using Protaper® endodontic system and Stainless steel hand K-files. The apical third was sealed using MTA and then the coronal two-thirds with warm gutta-percha (Figure 6).

**Figure 7:**
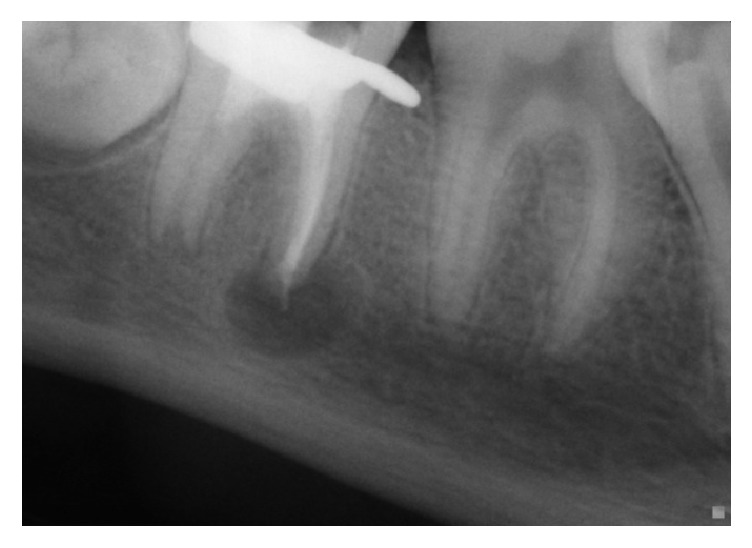
Radiograph 7 days after endodontic therapy.

In the next appointment, a coronal filling was performed using the closed sandwich technique with glass ionomer and composite (Figure 7).

**Figure 8:**
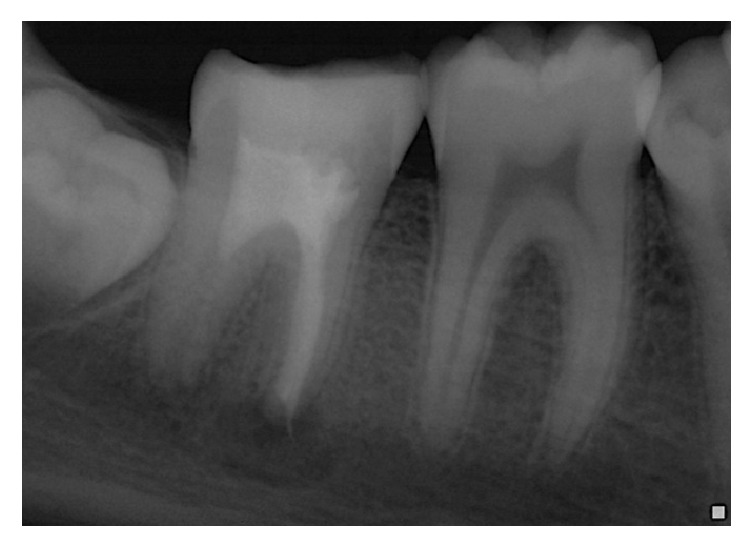
After 3 months from endodontic procedure on mesial roots: reduction of the radiolucent image at the mesial roots; healing of the periapical area at the distal root.

After the second procedure, the two-month recall showed a reorganization of the periodontal space of the mesial apex and important thickening of the distal root with a visible root edification and a thickening of the dentinal walls (Figure 8).

**Figure 9:**
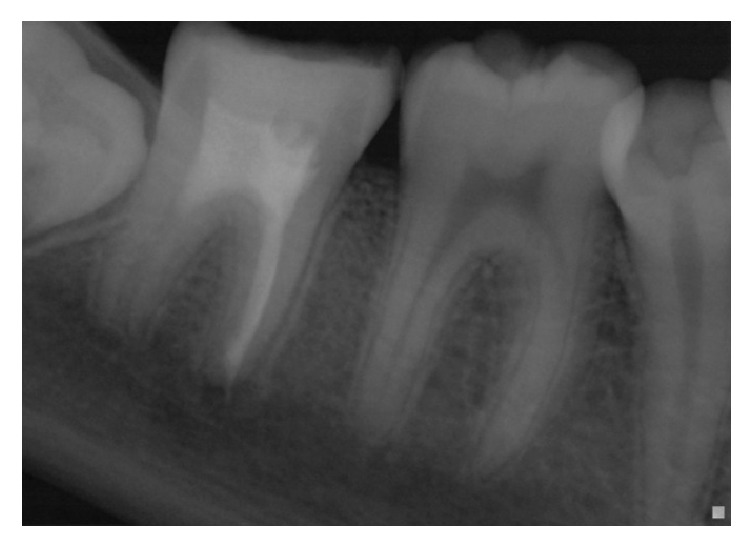
Radiograph at 24 months' recall: complete disappearance of the radiolucent images.

The 24-month recall (Figure 9) showed ad integrum periodontal regeneration at the apical area of the mesial roots, following the apexification procedure with MTA, and at the distal apical area with restructuring of the apical dome and thickening of the apical constriction, following the revascularization therapy.

## 6. Discussion

A range of clinical protocols have been described, with various irrigants, intracanal medication, clinical procedures, and follow-up times [8]. Criteria for predictable revascularization are still lacking. We have chosen in this clinical case to follow the protocol described by the AAE [9].

It is difficult to select the appropriate nonvital teeth with residual vital apical cells, which are believed to be necessary for a successful regenerative procedure [4, 8].

The natural regeneration ability of the dental pulp is widely used in dental practice. Indeed, the carious lesions cause necrosis of the odontoblasts in contact with the damaged dentin.

Magloire et al. [10] demonstrated that pulpal progenitor cells will migrate to the necrotic zone and differentiate into odontoblasts after controlling the initial inflammatory and immune response and then produce a reparative dentin, thus providing protection of the pulp tissue. This process showed that some cells in the adult dental pulp preserve their differentiation potential into odontoblasts [10].

The regenerative capacity of the pulpo-dentinal complex from pulp stem cells could be used in the tissue engineering approaches [5, 10, 11].

Nowadays, two types of tissue engineering are developed from the pulp. (1) The existence of pluripotent stem cells makes the tooth an interesting element with easy access to collect stem cells and it is considered in autologous therapies [12–14].

(2) Other applications include root canal revascularization, pulp implants, injections of hydrocolloids biogels in the root canal seeded with cells, or gene therapy in order to develop new endodontic therapies supplanting the conventional pulpectomy and canal obturation. [15–17].

The pulp capping and the regenerative procedure are used clinically, the other procedures must be verified and their interest confirmed regarding the existing techniques before including them in our therapeutic arsenal [16–18].

The patient must be informed that regenerative procedure is related to failure which could be related to the disappearance of clinical signs or either the immediate or delayed rewarming of the initial infection

The failure of revascularization therapy at the mesial root prompted us to reflect on the various causes.

When revascularization therapy is planned on a molar, bleeding control must be checked on each canal entry; in our clinical case it is possible that we had a nonbleeding of the mesial canals masked by the blood coming from the distal canal.

The viability of the stem cells at the mesial roots can be compromised by periapical infection. In this way, even if we had a bleeding, the clot would be lacking stem cells. These cells may exist but in insufficient number or potential for differentiation. Bansal et al. [19] discussed the possibility of long standing infection destroying the cells able to insure pulp regeneration. However, considering the successful outcomes of regenerative endodontic treatments in cases with long-lasting apical periodontitis, they concluded that this might not be the reason.

It can be deduced that this therapy is* patient-dependent*: more favourable in young patients with an important cell turnover;* practitioner-dependent*: requiring training and control of the procedure;* tooth-dependent*: can be used only for immature teeth. And following our clinical case, we can say that it is* canal-dependent*: influenced by the diameter of the foramen of the root and the viability of the apical stem cells [5, 6, 9, 13].

In our clinical case we had a radiological success in the distal root and a reinfection on the mesial; based on the principle of tissue economy and believing in the potential of periodontal regeneration, we opt for classic apexification with MTA which is a revolutionary material in endodontics. Since its introduction in the 1990s, several studies have demonstrated its use in many clinical applications. MTA has been extensively studied and is currently used for perforation repairs, apexifications, regenerative procedures, apexogenesis, pulpotomies, and pulp capping [3]; we used it on the mesial roots, retaining the therapeutic success of the distal root due to the healing of the apical periodontitis.

## 7. Conclusion

Endodontic regeneration techniques are promising and innovative for the treatment of immature teeth and could change our approach regarding endodontic procedure within the perspective of a minimally invasive dentistry.
